# The Rapid Forgetting of Faces

**DOI:** 10.3389/fpsyg.2018.01319

**Published:** 2018-07-27

**Authors:** Dana Krill, Galia Avidan, Yoni Pertzov

**Affiliations:** ^1^Department of Psychology, Hebrew University of Jerusalem, Jerusalem, Israel; ^2^Department of Psychology, Ben-Gurion University of the Negev, Beersheba, Israel; ^3^Department of Cognitive and Brain Sciences, Ben-Gurion University of the Negev, Beersheba, Israel

**Keywords:** forgetting, visual working memory, visual short-term memory, face inversion effect, face perception, face recognition

## Abstract

How are faces forgotten? Studies examining forgetting in visual working memory (VWM) typically use simple visual features; however, in ecological scenarios, VWM typically contains complex objects. Given their significance in everyday functioning and their visual complexity, here we investigated how upright and inverted faces are forgotten within a few seconds, focusing on the raw errors that accompany such forgetting and examining their characteristics. In three experiments we found that longer retention intervals increased the size of errors. This effect was mainly accounted for by a larger proportion of random errors - suggesting that forgetting of faces reflects decreased accessibility of the memory representations over time. On the other hand, longer retention intervals did not modulate the precision of recall – suggesting that forgetting does not affect the precision of accessible memory representation. Thus, when upright and inverted faces are forgotten there is a complete failure to access them or a complete collapse of their memory representation. In contrast to the effect of retention interval (i.e., forgetting), face inversion led to larger errors that were mainly associated with decreased precision of recall. This effect was not modulated by the duration of the retention interval, and was observed even when memory was not required in the task. Therefore, upright faces are remembered more precisely compared to inverted ones due to perceptual, rather than mnemonic processes.

## Introduction

Working memory refers to the short-term storage and manipulation of sensory information (Baddeley and Hitch, 1974). It is considered to be a core cognitive process underpinning a range of behaviors from perception to problem solving and action control (Hitch and Baddeley, 1976; Baddeley et al., 1985). Visual working memory (VWM) is involved in many perceptual and cognitive processes such as planning visually guided actions (Hayhoe et al., 2003; Hollingworth et al., 2008), however, it is highly limited. Its capacity limitations have been amply investigated and debated (Luck and Vogel, 1997, 2013; Cowan, 2001; Bays and Husain, 2008; Ma et al., 2014) but its temporal limitations have attracted much less attention. The most pertinent literature related to this issue has focused on the temporal robustness of VWM (e.g., Regan and Beverley, 1985; Magnussen et al., 1996), but has not elaborately examined the impact of extending the retention interval on memory performance (i.e., forgetting). The decline in performance after longer, as compared to shorter delays, reflects the loss of information due to imperfect maintenance processes, rather than imprecise encoding into memory or retrieval processes that are identical in all delay conditions. Thus, comparing performance following two different intervals enables us to isolate the effect of forgetting and maintenance processes from effects related to encoding and retrieval processes.

One of the first studies to address short-term visual forgetting following various delay intervals was conducted by Phillips (1974). Participants were asked to detect a change in two consecutive sets of checkerboard stimuli that either differed by one cell or were identical. Drops in performance during the first 600 ms were shown to reflect a loss of low-level sensory information (i.e., they were sensitive to small position translations of the whole stimulus), whereas the loss of information following longer delays was not sensitive to small position translation and therefore was considered to be driven by forgetting processes in more abstract short-term visual memory. The current study addresses the latter form of forgetting that takes place following retention intervals of a few seconds but not less than 1 s.

Not all studies have documented forgetting at this time scale. Recent studies by Ricker and Cowan (2010, 2014) found that only characters that were unfamiliar to participants (i.e., Hebrew letters shown to participants who were not Hebrew speakers) were forgotten, whereas familiar letters (i.e., English letters for participants who were English literates) were not forgotten. The authors concluded that forgetting is typically counteracted by a rehearsal process that is more effective in familiar stimuli that can be easily named. Thus, in the current study we have used unfamiliar stimuli that are hard to name. In addition, Ricker and Cowan (2014) found forgetting mainly in conditions involving simultaneous presentation of memory items compared to sequential displays, and that most time-based forgetting occurred between 1 and 6 s and not at longer delays. Therefore, we use parallel displays of memory array and retention intervals of a few seconds.

The above studies shed some light on rapid visual forgetting, but they do not address the *mechanism* involved in forgetting and failing to report the features of the previously displayed item correctly. One possibility is that following extended retention intervals, individuals are not able to access some of the memory representations that were accessible following shorter intervals. Alternatively, people may be able to maintain and access the object in memory but its representation becomes noisier and less precise with time.

To address this question, studies typically implement a delayed estimation task (Prinzmetal et al., 1998; Wilken and Ma, 2004; Zhang and Luck, 2008; Bays et al., 2009). In this paradigm, participants are required to reproduce a previously observed stimulus from an analog cyclic scale, such as the color of the corresponding item, by clicking on a color wheel. These tasks encourage participants to remember the fine details of an item, rather than its verbal tag and enable the documentation of the distribution of errors, thus providing data on the type of errors committed by participants. For example, a complete failure to access a memory representation should be manifested as a uniform distribution of errors across the scale, whereas a degradation in the fidelity of a representation should lead to a broader distribution of errors around the correct value.

Results obtained on these delayed estimation tasks suggest that extending the delay interval influences both types of errors: it increases the number of errors distributed randomly on the reporting scale, as well as broadens the distribution of errors around the correct target. In one study (Zhang and Luck, 2009), participants were shown displays with three simple objects (e.g., patches of color and shapes) and were required to reproduce one of the objects after a delay of 1, 4, or 10 s. Extending the retention interval led to a significant increase in random errors and to a modest, insignificant effect on the width of the distribution of errors around the correct feature of the item. A more recent study that used a larger variety of memory loads showed that more items in memory lead to steeper forgetting, which was reflected in both random errors as well as less precise reports (Pertzov et al., 2016).

Overall, these studies imply that when multiple, hard to verbalize visual objects are maintained in memory over extended time intervals, performance declines, as manifested in a greater number of random errors and more variable responses. Critically, all these studies addressed memory for simple features such as orientation, color, and simple shape elements. These stimuli, however, do not reflect the demands placed on the visual system in real life situations. Under such conditions, we hardly ever need to remember simple shapes and colors, but rather, we are required to remember complex objects such as the identity of the person in front of us in the line to a ticket booth. This distinction raises a critical question that has not been addressed to date; namely, how do people forget ecological (or more complex) objects? The present study focuses on memory for faces, given their unique ecological significance.

The way ecological, complex objects are maintained in memory may differ from the way basic features are maintained. Basic features are processed (Tong, 2003) and maintained (Harrison and Tong, 2009) in low-level visual cortex, whereas complex objects are processed and maintained in cortical regions higher up in the processing hierarchy (Grill-Spector and Malach, 2004). Indeed, the ability to remember complex objects in WM was shown to be more limited than the ability to remember simple features (Jiang et al., 2008). However, to the best of our knowledge, no study has reported how complex objects are forgotten across extended retention intervals. Two recent studies have used a delayed estimation task with face stimuli, but the employment of a fixed retention interval precluded the assessment of maintenance processes and forgetting. In one study (Lorenc et al., 2014), participants were asked to report the identity of a previously displayed face out of a set of 80 possible computer generated faces that varied continuously in terms of age and gender. The study showed that simply turning a set of faces upside-down lead to an increase in the width of the distribution of errors but did not modulate the fraction of random errors. Thus, this study suggests that memory representations of inverted faces are less precise (Lorenc et al., 2014). Another study (Zhou et al., 2018) investigated memory of faces of people from the same vs. other race with respect to the observer. They found that following long encoding time, the other race effect (ORE) was reflected in more random errors in the other race condition. When encoding time was more limited, other race faces were reported less precisely. The authors concluded that the ORE is driven by an inefficient encoding of other-race faces due to lack of visual experience with such faces.

As noted above, the usage of a fixed retention interval in these two studies did not enable them to isolate the effect of forgetting since reporting errors could be attributed also to visual perception, memory encoding and retrieval. Moreover, the usage of a limited face dataset in these experiments might have encouraged participants to attach verbal tags to the stimuli (e.g., the young man) and therefore confounded any direct assessment of VWM, a point which we further elaborate on in the section “Discussion”.

The present study investigated how faces are forgotten by using a delayed estimation task. One possibility is that faces are forgotten similarly to simple objects – hence their memory becomes less precise with time and sometimes becomes completely inaccessible. Alternatively, it could be that the precision of memory is stable and complex memory representations become inaccessible with time, or vice versa, that precision degrades with time but all representations stay accessible. To validate that the process we investigate is immediate forgetting of active representations we have used a large set of natural faces (with comparable age) that were displayed simultaneously. The use of a large set of stimuli, as opposed to a single set in all trials, is expected to hamper the usage of verbal and long-term memory strategies. This procedure, along with the incorporation of a delayed estimation task with various delay intervals enabled us to directly explore the mechanism behind immediate forgetting of complex objects from VWM for the first time.

## Experiment 1

### Methods

#### Participants

Twelve university students from the Hebrew University of Jerusalem, with normal or corrected-to-normal vision and normal color vision according to self-reports (mean age: 24.3 ± 1.8, eight female) participated in Experiment 1, which consisted of three 1-h experimental sessions. The study was approved by the Hebrew University ethics committee. All participants provided informed consent and received course credit or monetary compensation (∼$10.00 per hour).

#### Stimuli

One hundred and ninety realistic, color pictures of faces (78 female and 112 male) were taken from the following databases: Productive Aging Lab Face Database (Minear and Park, 2004), The IMM Face DB (Nordstrøm et al., 2004), and the Glasgow Unfamiliar Face Database (GUFD) (Burton et al., 2010). All faces had a neutral expression. The photos were cropped in a fixed round form, without hair (using Adobe Photoshop CS6). To study VWM and prevent verbal tagging and using long-term memory strategies, all faces were displayed only once on a given block and all faces in a trial had similar age, gender, skin tone and facial shape (e.g., cheekbones, jaw line). Each trial consisted of a circle of 18 faces (**Figure 2**) composed of three original faces from the pool and five morphed faces (Abrosoft FantaMorph deluxe V5) between each pair (83%A/17%B, 67%A/33%B, 50%A/50%B, 33%A/67%B, 17%A/83%B). Stimuli were presented on a 24-inch Dell U2412M monitor (resolution 1920^∗^1080) and participants were positioned at a viewing distance of 60 cm from the screen.

#### Procedure and Experimental Design

**FIGURE 1:**
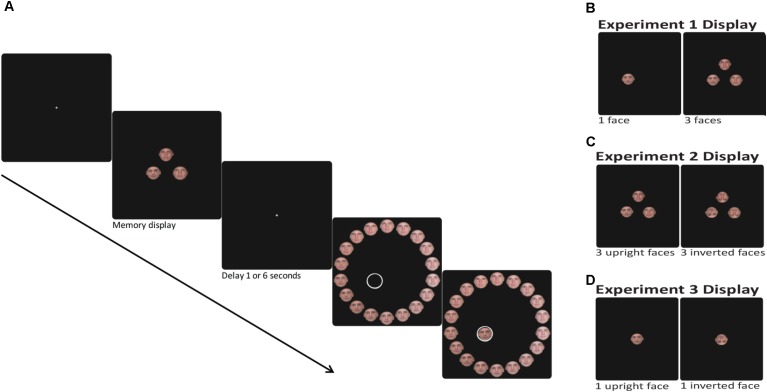
**(A)** Experimental design of one working memory trial. One or three faces (upright or inverted) are presented, followed by 1 or 6 s of a blank delay. Then, a spatial cue indicates which face from the memory array should be reported by selecting a face from the 18 face report circle. During the reporting stage the selected face appeared at the cued location. **(B)** The memory display of Experiment 1 consisted of one or three upright faces. **(C)** The memory display of Experiment 2 consisted of three upright or inverted faces. **(D)** The memory display of Experiment 3 consisted of one upright or inverted face.

**FIGURE 2:**
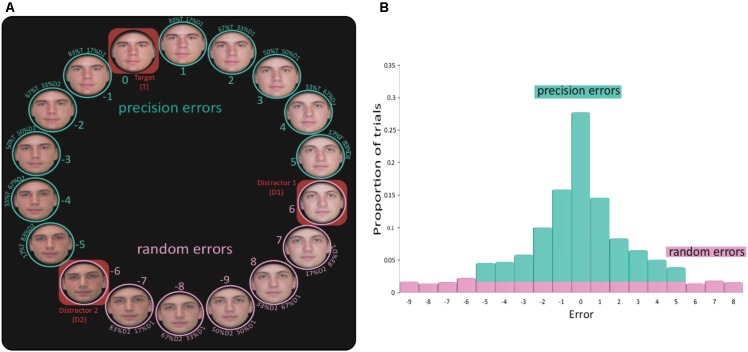
Analysis technique. **(A)** The report circle comprised of 18 faces. The three original (un-morphed) faces are marked by red rectangular frames. In this example, face ‘0’ is the target face on which the participant is required to report, as this is the correct answer. Faces 6 and –6 are the other two original faces, and all the faces between them are linear morphs between the two original faces. When the subject selected a face that includes any resemblance to the target face (–5 to 5), this was treated as a precision error. Selection of a face that had no resemblance to the target face (errors above 5 and below –5) was treated as a random error. **(B)** An example of an error distribution of one participant in one condition. Precisions errors are marked in green and random errors in pink.

The experiment was programmed in MATLAB and Psychophysics Toolbox (Brainard, 1997; Pelli, 1997). The segment of the experimental design common to all the experiments is illustrated in **Figure 1**. Each trial began with the presentation of a central fixation cross (white, 3 pixels, 0.08° of visual angle) for 1,000 ms. This was followed by a stimulus array consisting of one or three faces (each face picture was displayed in 200^∗^200 pixels, 5.17° × 5.17°). In trials with three faces in the memory array, faces were placed on the circumference of a circle with a radius of 150 pixels (3.88°) just above fixation and 120° clockwise and anticlockwise of the vertical meridian. In trials with one face in the memory array, the face was displayed randomly in one of those locations. The memory array was displayed on a black background for 1,500 and 4,500 ms for the 1 and 3 face conditions, respectively. Participants were instructed to remember the faces and, after a variable delay (1 or 6 s), to report the identity of one of the faces (the specific target face was cued by an empty circle at its original location). Participants reported the face identity by selecting a face from a circle of 18 faces [with a radius of 500 pixels (12.88°) around fixation; see illustration in **Figure 2**]. The circle was randomly rotated on every trial in order to hamper learning of the position of the original faces.

There were two types of trials distributed randomly among the three face conditions. In one, the 18 face report circle was composed of the target face and two novel faces. In the second type, the three faces from the memory array composed the report circle. The latter type of trials were harder because participants could misremember the exact location of the target face and erroneously report one of the other faces that appeared in the memory array (i.e., source error). In the former type, the two other faces that composed the reporting cycle were not related to the faces in the memory array, so participants were not likely to report the wrong face simply because of a confusion with a face from the memory array. We have used the two types of trials in order to control for the existence of such source errors in the experiment (i.e., reporting the wrong face due to misremembering of its location in the memory array). Previous studies have shown that such errors have significant contribution to forgetting of simple features such as orientation (Pertzov et al., 2016) and position (Pertzov et al., 2012). The chi-square analysis described below have validated that such confusion errors were observed in the condition in which the report circle consisted of the three faces in the memory array but not when it consisted of only one displayed face.

One block of the task included 30 trials consisting of all conditions in equal proportions: 10 trials with one face, and 20 with a three face array – 10 from each type of trials described above. Half of the trials had a 1-s delay, and half had a 6-s delay. None of the faces was repeated within a block and trials were randomly ordered within a block. Participants completed as many blocks as possible in an hour (between 4 and 6). To encourage participants to engage in the task, a feedback was presented every 10 trials, depicting the average error rate on the last 10 trials (the error rate calculation is described in the “Data Analysis” section). A score of 100 was given if the participant’s average magnitude of error was less than 1, and the score decreased with an increased error rate to a score of 60. The data of all experiments are available via the OSF at https://osf.io/t59p6/?view_only=37c9d58899774d8ba5615a9137716194.

#### Data Analysis

First, we analyzed the averaged size of absolute error when each report was considered with respect to the correct answer: error size was calculated as the distance between the participant’s answer and the correct target face. For example, participants were assigned an error score of zero if they selected the target face they were presented with. For an adjacent face to the target face (most similar morph), the error was 1 or −1 for clockwise or anticlockwise errors respectively. The errors on all trials of each participant and in each condition yielded a frequency distribution of errors (**Figure 2B**). To detach the analysis from any assumptions regarding the distribution of errors (Ma, 2018), we first analyzed the results using the mean absolute raw errors of each participant in each condition, similarly to earlier studies using a similar experimental procedure (Pertzov and Husain, 2013; Pertzov et al., 2013, 2017; Liang et al., 2016). Next we divided the distribution of errors to precision and random errors as illustrated in **Figure 2**.

Many of the studies that used delayed estimation tasks have used a data fitting procedure to dissociate the distribution to two or three underlying components (Zhang and Luck, 2009; Lorenc et al., 2014; Pertzov et al., 2016). However, this approach was less appropriate here because the distribution of errors is not likely to be smooth and cyclic as in the feature domain. While in other stimulus domains the similarity of the items in the reporting scale gradually changes across the scale, in this experiment only part of the stimuli were similar to the target object (all morphs that included the target face) and other stimuli were completely different than the target item (the two non-target original faces and the morphs between them). Hence, we explored the different types of errors directly using the following approach: We extracted two summary statistics from each distribution of errors: (1) **Proportion of random errors:** when a participant chose a face from the circle that did not have any resemblance to the target face [morph did not include any fraction of the target face (errors 6,7,8,9 in absolute value)]. In such cases we assumed that the participant did not remember the target face, and therefore just guessed. To obtain a proportion value, the number of such errors was divided by the overall number of trials. (2) **Precision of recall** – is calculated based on trials in which a participant reported a face that had some resemblance to the target face (i.e., was a morph of the target face). In such cases, we assumed that participants had some recollection of the target face. To quantify the degree of recall precision, we averaged the magnitude of the absolute errors from 0 to 5. Note that when participants did not have any recollection of the target face, they were likely to guess a random face and therefore sometimes report a face with some resemblance to the target face. Therefore, the number of random errors per bin (average number of errors of 6,7,8,9 in absolute value) was subtracted from the precision errors, and added to the proportion of random errors (see uniform distribution in **Figure 2**).

Note that the two summary statistics: (1) Proportion of random errors and (2) Precision of recall, are somewhat independent of each other. The proportion of random errors is sensitive only to the proportion of trials defined as random while precision of recall is sensitive to the *magnitude* of errors which is related to the shape of the error distribution rather than to the proportion of trials in it. Thus, it is reasonable that an experimental manipulation would modulate the proportion of random errors but not the precision of recall, and vice versa.

For statistical analysis, we applied a repeated measures ANOVA with number of faces (1 or 3) and delay duration (1 or 6 s) as factors. The mean absolute errors, the proportion of random errors and the average precision errors were the dependent variables (three different mixed ANOVAs). We also subjected the data to a JZS Bayes factor ANOVA (Rouder et al., 2012; Love et al., 2015; Morey and Rouder, 2015). Whereas a typical analysis of *p*-values does not enable the interpretation of null effects, this Bayesian technique allowed the evaluation of the strength of the evidence in favor of the null effect. In the main text we report the two models with the highest posterior probability and the probability ratio between them. A table with the full set of Bayes Factors (BFs) is provided in the Supplementary Materials – 1.

We used chi-square analysis to test whether two distributions were significantly different with no assumption on the type of distribution of errors. Because chi-square analysis requires a large number of samples per bin (Armitage et al., 2002), we collapsed the distributions of all participants into a single distribution.

First, we used chi-square to complement our comparisons between the average precision errors by comparing the entire distributions of precision errors (we used errors of 5 and below) rather than comparing a single summary statistic.

We also used chi-square analysis to validate our assumption about the uniform distribution of random errors. As mentioned above, we assumed that if a participant did not remember the target face, this constituted a guess and therefore the distribution of guesses should not be different from a uniform distribution. For the purpose of validation, we ran a chi-square to compare the distribution of errors above 5 to a theoretical uniform distribution. Note that unlike the chi-square analysis of precision errors in which *two* empirical distributions were compared, all the random distributions were expected to be uniform. Therefore, in the random error distributions, we did not compare two empirical distributions (both were assumed to be uniform) but rather each distribution to a theoretical uniform distribution to validate our assumption. In most of the conditions the distributions of random errors (>5) seem to spread uniformly [all χ^2^(3) < 6.943, all *p* > 0.05]. However, the distribution of errors in the condition in which the three faces from the memory array composed the report circle was significantly different from a uniform distribution [χ^2^(3) = 15.89, *p* ≤ 0.001]. This is expected since these trials were harder and participants might have misremembered the exact location of the target face and reported one of the other faces from the memory array (i.e., source error). In fact, in this condition the distribution of random errors deviated from uniformity because participants tended to select the other two original faces in the circle (error of 6 and -6) more than the other errors defined as random (>6) [paired one-tailed *t*-test (10) = 1.825, *p* = 0.049]. Because the focus of this study was not on source errors but rather on the rate of forgetting and its relationship to random and precision errors, we excluded this condition from the remainder of the analyses reported in the main text. Further analyses and a discussion of source errors in this condition are described in the Supplementary Materials – 2. Note that including this condition in the analysis did not lead to qualitative changes in the results.

### Results

We first calculated the **mean raw error**: the absolute values of the errors of each participant and each condition were averaged. A repeated measures ANOVA confirmed that the general mean (absolute) error rate increased with the increase in set size [Set size main effect: *F*(1,11) = 19.103, *p* ≤ 0.001, η^2^ = 0.635]. There was a descriptive increase in error rate with an increase in delay duration [Delay main effect: *F*(1,11) = 4.231, *p* = 0.064, η^2^ = 0.278], and no significant interaction [interaction: *F*(1,11) = 0.463, *p* = 0.51, η^2^ = 0.04].

A JZS Bayes factor ANOVA with a default prior supported these results. The model, including the two main effects of delay and set size, yielded the highest Bayes factor and was more probable than all the other models (BF 2 main effects without interaction = 3.458 vs. BF Set size main effect = 2.092, BF-Ratio: 1.652).

Next, out of each error distribution of a specific condition and subject, we extracted two summary statistics: (1) **Random errors** as a proportion, and (2) **Precision of recall**: the average magnitude of the precision errors (error-size ≤ 5).

#### Random Errors

First, we verified that the large errors (>5) we refer to as random were indeed uniformly distributed. Chi-square analysis confirmed that all the distributions (two delays and two set sizes) were not significantly different from the expected uniform distribution (*p* > 0.05 for all conditions).

**FIGURE 3:**
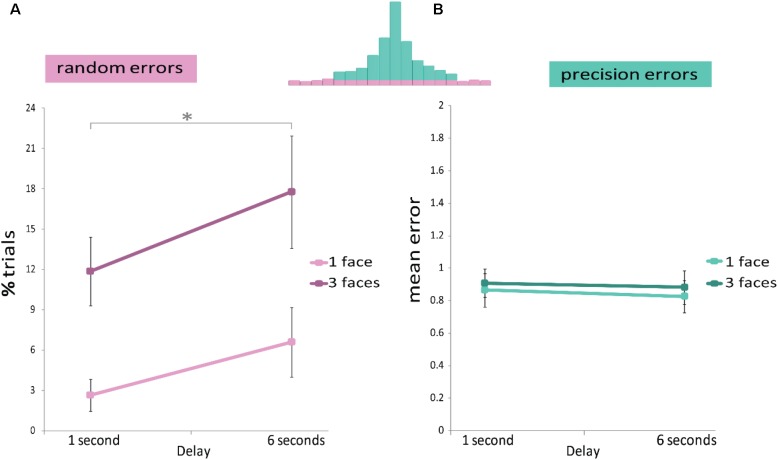
Results of Experiment 1. The memory display consisted of one or three upright faces. A repeated measures ANOVA analysis showed that **(A)** Random errors were modulated by delay duration (*x*-axis) and set size (light and dark pink). **(B)** Precision errors were not affected by either delay duration (*x*-axis) or set size (light and dark green). *^∗^p* < 0.05.

A repeated measures ANOVA (**Figure 3A**) confirmed that the proportion of random errors increased with delay duration [Delay main effect: *F*(1,11) = 6.286, *p* = 0.029, η^2^ = 0.364], as well as with the increase in set size [Set size main effect: *F*(1,11) = 19.437, *p* ≤ 0.001, η^2^ = 0.639]; but with no significant interaction [interaction: *F*(1,11) = 0.524, *p* = 0.484, η^2^ = 0.045]. This analysis indicates that longer delays as well as increased memory load increase random errors but the two do not interact.

A JZS Bayes factor ANOVA (Rouder et al., 2012; Love et al., 2015; Morey and Rouder, 2015) with a default prior supported these results. The model including the two main effects of delay and set size yielded the highest Bayes factor and it was more probable than all the other models (BF 2 main effects without interaction = 5.98 vs. BF 2 main effects + interaction = 1.274, BF-Ratio: 4.7).

#### Precision Errors

A repeated measures ANOVA (**Figure 3B**) showed no effect of delay duration or set size, or interaction [Set size main effect: *F*(1,11) = 1.414, *p* = 0.259, η^2^ = 0.114. Delay main effect: *F*(1,11) = 0.417, *p* = 0.532, η^2^ = 0.037. Interaction: *F*(1,11) = 0.015, *p* = 0.904, η^2^ = 0.001].

Consistent with the results above, a JZS Bayes factor ANOVA on precision errors revealed that the null effect model was more probable than all the other possible models (BF Null effects = 4.117 vs. BF Set size main effect = 1.117, BF-Ratio: 3.7).

The chi*-*square statistic supported these results: the distributions underlying precision errors were not significantly different for the 1 and 6 s delays [χ^2^(10) = 13.386, *p* = 0.203]. Unlike the ANOVA analysis on the average magnitude of the precision errors (summary statistics of the distribution), the *distributions* of errors seemed to be different when one or three faces were displayed [χ^2^(10) = 20.558, *p* = 0.024]. We elaborate on this in the section “Discussion”.

### Discussion

To study forgetting of complex objects we used a delayed estimation task with images of natural color faces, all with comparable age and gender. We found that longer delays increased the averaged size of errors which was reflected mainly in random errors but not in the precision of recall. Moreover, increased memory load led to larger errors accompanied by larger proportion of random errors, and a slight change in the distribution of precision errors (as captured by chi-square analysis) but did not change the averaged size of precision error (as captured by an ANOVA on the averaged precision error). Despite the large difference between simple features and complex objects, such as faces, these findings are somewhat consistent with the results obtained with tasks employing simple features (Zhang and Luck, 2009; Pertzov et al., 2016). Thus, when complex objects are forgotten their representation is rendered inaccessible and reflected in random errors. However, in contrast to the case of simple features (Pertzov et al., 2016), the precision of recall does not seem to degrade in longer retention intervals.

## Experiment 2

Experiment 1 explored immediate forgetting of faces, under low and high memory loads (one or three faces). A wealth of behavioral literature posits that faces are processed in a qualitatively different fashion compared to other visual categories (Rossion, 2013). Hence, a critical question is whether the pattern of forgetting we observed in Experiment 1 is unique to faces and their form of processing, or alternatively, could be generalized to other complex objects. One hallmark of face specific processing mechanisms is the face inversion effect (Yin, 1969), that is, the disruption of face processing due to inversion compared to the effect of this manipulation on other objects. Inverted faces provide an ideal stimulus to employ in our experiment as their low level image properties are identical to those of upright faces yet, their processing is markedly different compared to upright faces (Yin, 1969; Rossion and Gauthier, 2002; Richler and Gauthier, 2014). Thus, in Experiment 2 we directly compared the forgetting of upright and inverted faces.

### Methods

#### Participants

Forty-two university students from the Hebrew University of Jerusalem, with normal or corrected-to-normal vision and normal color vision according to self-reports (mean age: 24 ± 4.5, 19 female) participated in Experiment 2, which consisted of a 1-h experimental session. Note that in the previous experiment participants underwent three 1-h sessions. In order to decrease the number of repetitions of the same face images along the experiment, which might lead to the specific labeling of faces, we chose to test a more participants using a single testing session.

The study was approved by the Hebrew University ethics committee. All participants provided informed consent and received course credit or monetary compensation (∼$10.00). None of the participants in Experiment 2 participated in Experiment 1. Four participants were excluded from the analysis because their average error was more than 2 SD above the mean across participants. Inspection of their error distribution suggested that they did not follow instructions and reported random faces even in the easiest condition.

#### Stimuli

The stimuli were identical to those used in Experiment 1 except for a single change. To make sure that participants were not able to recognize the original faces among the morphed faces due to some high frequency features that are lost in the morphing process, we blurred all faces (morphed and original) using Microsoft Office Picture Manager and reduced the contrast by 20 units.

#### Procedure and Experimental Design

The procedure was similar to Experiment 1, except that the memory display consisted of three upright or three inverted faces, followed by a circle of 18 upright or inverted faces, respectively (**Figure 1C**).

Blocks consisted of 30 trials. For half of the participants, each block consisted of all conditions (half with a 1-s delay, half with a 6-s delay). Each trial was randomly assigned to the upright or the inverted condition. For the other half of the participants, upright and inverted faces were used in different blocks, with equal proportions of delay conditions within a block. Trials were displayed in random order. None of the faces was repeated within a block. Participants completed as many blocks as possible in an hour (between 4 and 6).

#### Data Analysis

The analysis was similar to Experiment 1. We applied a three factor mixed ANOVA with face orientation (upright or inverted), and delay duration (1 or 6 s) as within subject factors, and experiment type (mixed blocks or uniform blocks) as the between subjects factor. The mean absolute errors, the proportion of random errors and average precision errors were the dependent variables (three different mixed ANOVAs). Experiment type (mixed or uniform blocks) had no effect (main or interaction) on random or precision errors. Therefore, for better readability, the non-significant effects of experiment type are not reported in the results section (but this factor was still included in the mixed ANOVA). As in Experiment 1, we also conducted Bayes factor and chi-square statistical analyses.

### Results

A mixed ANOVA confirmed that the general mean (absolute) error increased with delay duration [Delay main effect: *F*(1,36) = 6.364, *p* = 0.016, η^2^ = 0.124], as well as with face orientation [Upright-Inverted main effect: *F*(1,36) = 60.345, *p* < 0.001, η^2^ = 0.618], but there was no significant interaction [interaction: *F*(1,36) = 0.906, *p* = 0.347, η^2^ = 0.024].

A JZS Bayes factor ANOVA with a default prior supported these findings. The model including the face orientation main effect yielded the highest Bayes factor and was more probable than all the other models (BF Face orientation main effect = 8.083 vs. BF 2 main effects without interaction = 3.774, BF-Ratio: 2.1).

#### Random Errors

First, we examined whether large errors (>5) were uniformly distributed. Chi-square analysis confirmed that none of the distributions (two delays and two conditions) was significantly different from the expected uniform distribution (*p* ≥ 0.175 for all conditions).

**FIGURE 4:**
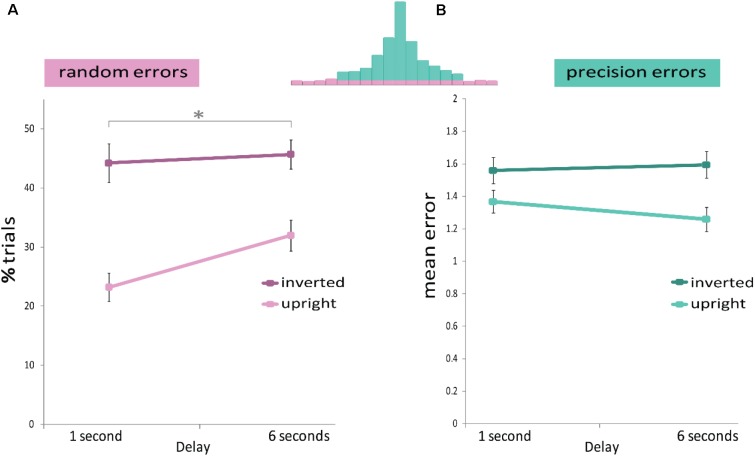
Results of Experiment 2. The memory display consisted of three upright or three inverted faces. A mixed design ANOVA revealed that **(A)** Random errors were modulated by delay duration (*x*-axis) and by face orientation (light and dark pink). **(B)** Precision errors were not modulated by delay duration (*x*-axis), but only by face orientation (light and dark green). *^∗^p* < 0.05.

A mixed ANOVA (**Figure 4A**) confirmed that the proportion of random errors increased with delay duration [Delay main effect: *F*(1,36) = 9.503, *p* = 0.004, η^2^ = 0.192], as well as with face orientation [Face Orientation main effect: *F*(1,36) = 45.425, *p* ≤ 0.001, η^2^ = 0.542], but there was no significant interaction [interaction: *F*(1,36) = 2.021, *p* = 0.164, η^2^ = 0.05]. These results indicate that longer delays, as well as face inversion, increase random errors, but the two factors do not interact.

A JZS Bayes factor ANOVA with a default prior supported these findings. The model including the two main effects of delay and face orientation yielded the highest Bayes factor and was more probable than all the other models (BF 2 main effects without interaction = 5.855 vs. BF 2 main effects + interaction = 3.199, BF-Ratio: 1.8).

#### Precision Errors

A mixed ANOVA (**Figure 4B**) revealed that precision errors increased when faces were inverted [Face Orientation main effect: *F*(1,36) = 20.765, *p* ≤ 0.001, η^2^ = 0.361]. However again, as in Experiment 1, no difference was found in precision errors between the two delay durations [Delay main effect: *F*(1,36) = 0.338, *p* = 0.565, η^2^ = 0.008]. The two factors did not interact [interaction: *F*(1,36) = 1.377, *p* = 0.248, η^2^ = 0.037].

The ANOVA results were consistent with the JZS Bayes factor ANOVA, that showed that the model including a main effect of face orientation was more probable than all other possible models (BF Face Orientation main effect = 17.421 vs. BF 2 main effects = 1.93, BF-Ratio: 9).

The chi*-*square statistics were consistent with the above results: the distributions underlying precision errors did not significantly differ for the 1- and 6-s delays (across object type) [χ^2^(10) = 13.895, *p* = 0.178]. The precision error distribution was significantly different when participants saw upright vs. inverted faces (across delays) [χ^2^(10) = 49.507, *p* < 0.001].

### Discussion

To further examine whether the forgetting pattern in Experiment 1 was specific to faces, Experiment 2 used inverted faces. As in Experiment 1, longer delays increased random errors but did not have an effect on precision errors. Moreover, inverting the faces, thus hampering face-specific processing mechanisms, led to larger errors accompanied by more random and larger precision errors. Thus, these findings indicate that the results obtained in Experiment 1 were not specific to upright faces.

## Experiment 3

Inverting a face alters its perceptual processing; therefore, it is not clear whether the differences between upright and inverted faces in Experiment 2 were due to perceptual or WM processes. To investigate whether the inversion effect was due to a failure in WM *per se* or alternatively in visual perception, Experiment 3 incorporated a condition in which the stimuli to be reported were presented simultaneously with the report circle. In this experiment, we used a single face in the memory array, since using multiple items leads to an inherent difference between the perceptual/simultaneous condition and the memory conditions. In the simultaneous condition, participants can look exclusively at the face to be probed and discard all other faces in the array. However, in the memory conditions all items in the memory array need to be processed. Therefore, in order to make the memory and perceptual conditions as similar as possible, we used a memory array of a single face (**Figure 1D**). Thus, in Experiment 3 we compared the reports of a single upright and inverted face, in simultaneous presentation and after two different delay durations.

### Methods

#### Participants

Twenty university students (mean age: 23.4 ± 3.1, 12 female) participated in Experiment 3, which consisted of a 45-min experimental session. None of the participants in Experiment 3 participated in Experiments 1 or 2.

#### Stimuli

The stimuli were similar to those used in Experiment 2. In Experiment 3 the stimuli were displayed at a viewing distance of 50 cm on a 17.3-inch Dell FDH (1920^∗^1080) Truelife LED-Backlit Touch Display.

#### Procedure and Experimental Design

The procedure was similar to the procedure in Experiment 2, with a few changes. In Experiment 3, the stimulus array consisted of one upright or inverted face (each face picture was in 200^∗^200 pixels, 4.5° × 4.5°) displayed in the center of the monitor, followed by a report circle of 18 upright or inverted faces, respectively [located on an imaginary circle around fixation with a radius of 470 pixels (10.6°)]. To allow sufficient time but to prevent participants from dwelling on this stage for too long, the reporting stage was limited to 20 s. If the participant did not press the space bar on time, the last face that the participant selected was recorded and the next trial began. This occurred in 1.3 and 4% of the trials on the memory and perception tasks, respectively; see explanation about memory and perception tasks in the following paragraphs. A touch screen was used for reporting the remembered face.

Each block was composed of 30 trials consisting of all conditions (half with a 1-s delay and half with a 6-s delay. Each trial was randomly assigned to the upright or the inverted condition). Trials were displayed in random order and none of the faces was repeated within a block. Participants completed three blocks of the memory task.

**FIGURE 5:**
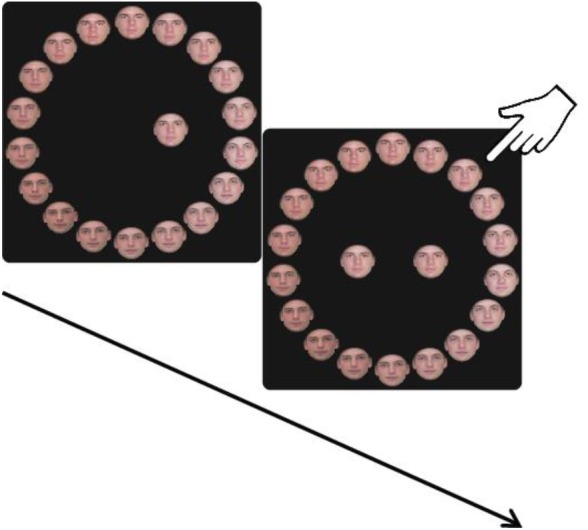
Experimental design of a perception trial. One face (upright or inverted) is presented, and at the same time, participants are asked to select the identical face out of an 18 face report circle (of upright or inverted faces, respectively). The selected object appeared at the center of the circle next to the displayed face.

Following the first memory block all participants were administered one perception block. The perception block consisted of 30 trials in which a single face was presented simultaneously with the circle of 18 faces. Participants were instructed to report the identity of the face (**Figure 5**).

#### Data Analysis

The analysis was similar to Experiment 1, except for the addition of the simultaneous condition. In the statistical analysis of memory and precision tasks, we applied a repeated measures ANOVA with face orientation (upright or inverted) and delay duration (*simultaneous*, 1 or 6 s) as factors. The mean absolute errors, the proportion of random and average precision errors were the dependent variables (three different repeated measure ANOVAs). The Tukey (HSD) test was used for *post hoc* pair-wise comparisons. Similar to Experiments 1 and 2, we also conducted a Bayes factor and a chi-square statistical analysis.

### Results

As in Experiment 2, a repeated measures ANOVA confirmed that the general mean (absolute) error rate increased with delay duration (three different delays: simultaneous – 0 delay, 1- and 6-s delays) [Delay main effect: *F*(2,38) = 62.605, *p* < 0.001, η^2^ = 0.767], as well as with face orientation [Upright-Inverted main effect: *F*(1,19) = 83.842, *p* ≤ 0.001, η^2^ = 0.815], but there was no significant interaction [interaction: *F*(2,38) = 0.492, *p* = 0.615, η^2^ = 0.025]. A *post hoc* Tukey test revealed that larger errors were committed in the 6-s delay as compared to the 1-s delay (*p* = 0.002), and the simultaneous presentation (*p* < 0.001), and larger errors in the 1-s delay as compared to the simultaneous presentation (*p* < 0.001).

A JZS Bayes factor ANOVA with a default prior supported these results. The model including the two main effects of delay and face orientation yielded the highest Bayes factor and it was more probable than all the other models (BF 2 main effects without interaction = 19.665 vs. BF 2 main effects + interaction = 0.814, BF-Ratio: 24.1).

#### Random Errors

First, we examined if the large errors (>5) were indeed uniformly distributed. Chi-square analysis of the memory task confirmed that none of the distributions (two delays and two conditions) was significantly different from the expected uniform distribution (*p* > 0.1 for all conditions). This chi-square analysis was not performed on the perception task because the number of trials with large errors did not reach the minimum number of samples per bin required by the chi-square analysis (5 per bin) (Armitage et al., 2002).

**FIGURE 6:**
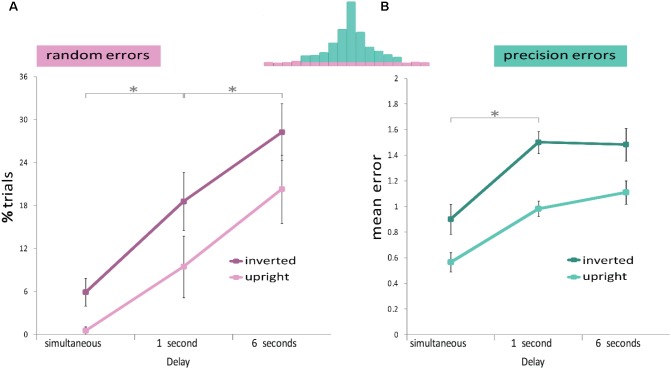
Results of Experiment 3. Reports of one upright or one inverted face after two delay conditions and a simultaneous display. **(A)** Random errors were modulated by delay duration (*x*-axis) and by face orientation. The effect of orientation was similar at all delays, including the simultaneous condition. **(B)** Precision errors were larger in the 1 and 3 s delay conditions with respect to the simultaneous condition. Precision errors were larger when the face was inverted, in all delay conditions. Error bars represent SEM across subjects. *^∗^p* < 0.05.

As in Experiment 2, a repeated measures ANOVA confirmed that the proportion of random errors increased with delay duration (three different delays: simultaneous – 0 delay, 1- and 6-s delays) [Delay main effect: *F*(2,38) = 17.49, *p* < 0.001, η^2^ = 0.479], as well as with face inversion [Face Orientation main effect: *F*(1,19) = 24.028, *p* ≤ 0.001, η^2^ = 0.558] but there was no significant interaction [interaction: *F*(2,38) = 0.366, *p* = 0.696, η^2^ = 0.019] (**Figure 6A**). A *post hoc* Tukey test revealed that more random errors were committed in the 6-s delay than in the 1-s delay (*p* = 0.017), and the simultaneous presentation (*p* < 0.001), and more random errors in the 1-s delay compared to the simultaneous presentation (*p* = 0.012).

These results indicate that longer delays, as well as face inversion, increased the proportion of random errors, but the two do not interact. Hence, the effect of delay was not different between inverted and upright faces. A JZS Bayes factor ANOVA with a default prior supported these results. The model including the two main effects of delay and face orientation yielded the highest Bayes factor and was more probable than all the other models (BF 2 main effects without interaction = 20.471 vs. BF 2 main effects + interaction = 0.584, BF-Ratio: 35).

#### Precision Errors

A repeated measures ANOVA showed that precision errors were larger when faces were inverted [Face Orientation main effect: *F*(1,19) = 80.99, *p* ≤ 0.001, η^2^ = 0.81], and when the delay was extended [Delay main effect: *F*(1,38) = 37.819, *p* < 0.001, η^2^ = 0.666] (**Figure 6B**). No significant interaction was observed [interaction: *F*(1,38) = 0.924, *p* = 0.406, η^2^ = 0.046]. However again, as in Experiments 1 and 2, when omitting the simultaneous condition, no difference was observed when extending the duration of the delay from 1 to 6 s [*post hoc* Tukey, *p* = 0.724]. A *post hoc* Tukey test revealed that precision errors increased from the simultaneous to the 1-s delay duration (*p* < 0.001) and from the simultaneous to the 6-s delay duration (*p* < 0.001).

A JZS Bayes factor ANOVA for precision estimates was consistent with the above results. When adding the perception task to the analysis, the model including two main effects was more probable than all the other possible models (BF 2 main effects without interaction = 14.614 vs. BF 2 main effects + interaction = 1.095, BF-Ratio: 13.3). Without the perception condition, the model that only included face inversion was more probable than all the other possible models (BF Face Orientation main effect = 9.693 vs. BF 2 main effects = 1.014, BF-Ratio: 9.5).

The chi*-*square statistic was also consistent with the above results and showed that the distributions of precision errors differed across delay durations when all three delays were considered [χ^2^(10) = 11.748, *p* < 0.001], but not when the simultaneous perception condition was discarded [χ^2^(10) = 18.102, *p* = 0.053]. The distributions of precision errors differed in the upright and inverted conditions [χ^2^(10) = 78.662, *p* < 0.001], and this difference was also evident without the simultaneous perception condition [χ^2^(10) = 65.22, *p* < 0.001].

### Discussion

To study whether the difference between memory reports of upright and inverted faces and the forgetting pattern found in Experiments 1 and 2 were due to WM processes or visual perception, we added a simultaneous presentation condition. Similar to the results of Experiment 2, but using a single face, longer retention intervals increased random errors but did not significantly influence precision errors. Moreover, inverted faces yielded a greater number of random errors and larger precision errors in all delay conditions, including the simultaneous perceptual condition when no memory processes were required.

The simultaneous perception task revealed that the differences between upright and inverted faces in random and precision errors were apparent even when the task had no memory requirements. This suggests that the inversion effect found in the memory reports was likely to be due to perceptual, rather than mnemonic processes (as claimed by Lorenc et al., 2014; see section “General Discussion”). Importantly, the differences revealed in the perception task could not account for the increase in random errors over time between 1- and 6-s delays. Any processes related to visual perception, memory encoding and retrieval should have exerted a similar effect on the 1- and 6-s delay conditions. Therefore we conclude that imperfect maintenance processes lead to increased random errors.

## General Discussion

We investigated how upright and inverted faces are forgotten within a few seconds. In all three experiments, we found that longer delays increased the averaged error by increasing the number of random errors but not by decreasing the precision of recall. Greater memory load and face inversion increased the number of random errors, but did not influence the rate of forgetting. Or to put it more intuitively, our chance to correctly recall a face decreases along a few seconds after we perceive it. Importantly, this forgetting pattern seems to be ‘all or none’, thus implying that we would not be able to access the item in memory at all. Inverting the faces led to decreased precision of recall but this decrease was also observed in the simultaneous condition and did not increase across delays, implying that this effect could be accounted for by perceptual processes (Experiment 3). Unlike precision errors, the increase in random errors over time could only be accounted for by time dependent memory degradation since these errors were more frequent in longer than in shorter delays. Hence, unlike the case of simple features, immediate forgetting of complex objects and specifically of faces, is reflected in random errors but not in decreased precision. Note that the current design and analysis is different in several aspects from the ones previously used in studies of VWM of simple features. Therefore, any direct comparison between forgetting of faces and forgetting of simple features requires further experiments that are carefully matched across the two stimulus types and analysis procedure.

### Forgetting of Faces

In three experiments, we found that forgetting of faces was reflected in a complete failure to access the representation of these faces (reflected as random errors) rather than noisier reports. Thus, unlike simple features which become noisier with time (Pertzov et al., 2016), forgetting of faces manifests as increased proportion of random errors. Random errors can be the result of “sudden death” (Zhang and Luck, 2009) of the memory representation or a failure to access a still-present memory representation. We do not, and cannot, distinguish between these two possibilities. In fact, theoretical and empirical studies of forgetting have claimed that one cannot prove that any information is completely forgotten, but simply that it could not be retrieved in the context investigated (Smith and Vela, 2001). Regardless of the exact reason for a complete failure to access memory representations, our experiments show that extended retention intervals decrease the probability that the face would be accessed rather than broadening the distribution of errors. This study is the first to highlight the apparent difference between the type of errors that accompany forgetting of simple and more complex objects, specifically faces. How is it that simple features become noisier with time but faces, which consist of many simple features, do not? We can only speculate that the “glue” that binds all the simple features and makes them a face is fragile and degrades in extended retention intervals. Once the features are no longer bounded together, the cues used to access the memory representation of the face could not be accessed anymore – leading to guesses. This view echoes previous findings showing that binding of object-to-location (Pertzov et al., 2012) and orientation-to-color (Pertzov et al., 2016) are fragile and degrade with extended retention intervals.

One could argue that these conclusions are based on null findings; namely, that p statistics are not appropriate for concluding that precision of recall do not differ as a function of the delay. To address this concern we also used a Bayesian inference technique that allowed us to evaluate the strength of evidence behind the two alternatives that precision of recall is influenced or not by the length of the delay. Additionally, we used chi-square analysis that avoids assumptions about the distribution of errors or the use of a summary statistic such as the average error (Ma, 2018).

### Delayed Estimation of Faces

To the best of our knowledge, this study is the first to investigate how faces are forgotten across retention intervals at the scale of seconds. However, a recent study by Lorenc et al. (2014) used the delayed estimation task to study how faces are remembered (but not forgotten, since they used a single delay). Consistent with the current study, Lorenc et al. (2014) found that inverted faces are remembered less precisely than upright faces at every set size. However, in contrast to our findings, they found no differences in the proportion of random errors between inverted and upright faces.

In relation to this issue we note that our simultaneous condition in Experiment 3 shed new light on the main finding of Lorenc et al. (2014). They have concluded that expertise for upright faces lead to more precise memory representations. However, the current study shows that improved precision is observed also in the simultaneous condition in which no mnemonic processes are required, thus supporting another notion, that is, that *perceptual processes* are more precise and efficient due to expertise but not mnemonic processes. In fact the forgetting slopes of inverted and upright faces were found to be equivalent – suggesting that memory maintenance is similar in both cases.

The conclusions that can be drawn from the Lorenc et al. (2014) study about the way faces are forgotten from WM are somewhat limited. First, these authors used a single duration of delay and therefore could not address forgetting (defined as the loss of performance across extended delays), and could not determine whether differences were due to perceptual or mnemonic processes. Second, a single database of artificial faces was repeated across all trials in their experiment. Thus, the stimuli were likely to become familiar with time, enabling participants to use long-term memory strategies rather than purely working memory when performing the task. Such repetition may encourage participants to use verbal tags to remember the objects (e.g., code a face as an “elderly man” etc.). Previous studies have shown that the memory of object identity is strongly modulated by participants’ ability to assign a verbal label to objects. Even when shapes were hard to name but the same shapes were frequently repeated, participants were able to attach labels to the shapes and counteract forgetting via rehearsal (Simons, 1996). The third limitation of the design (Lorenc et al., 2014) was that the array of faces was presented in a sequential manner, one face at a time. Sequential presentation of stimuli, however, can potentially encourage rehearsal and may result in slower forgetting compared to simultaneous presentation of stimuli (Ricker and Cowan, 2014). Accordingly, Lorenc et al. (2014) experiment led to very few random errors, therefore it is not surprising that they did not reveal any significant differences related to this measure.

In the current study, each trial within a block consisted of a new set of real faces (i.e., not computer generated) which were displayed simultaneously. Hence, it was hard for participants to use verbal and long-term memory strategies. These steps, in addition to the employment of different delay intervals, enabled us to directly explore rapid forgetting of faces from VWM. Thus, our experimental design was tailored to study VWM of faces and, unlike Lorenc et al. (2014), was able to elicit a significant number of random errors. Given our careful design, we were able to probe VWM and reveal that the frequency of random errors was clearly elevated when faces were inverted and retention interval was extended, thus demonstrating that these manipulations influence the probability of accessing previously encoded items.

Most previous studies have used binary responses (such as ‘yes/no’ or ‘same/different’; but see Lorenc et al., 2014; Zhou et al., 2018). In contrast, the current task provides a way to explore different types of errors by distinguishing between random errors and precision errors that are clustered around the target item. We report here, for the first time, that when faces are forgotten, their memory representation does not become noisier but rather it becomes less accessible to conscious report, presumably due to the collapse of the binding between the various features of the face. We believe that this task can contribute to various other fields of research, for instance, exploring the type of errors committed by individuals with prosopagnosia, to help better characterize their visual impairment.

## Author Contributions

DK, GA, and YP designed the experiments and wrote the manuscript. DK performed the experiments and analyzed the data.

## Conflict of Interest Statement

The authors declare that the research was conducted in the absence of any commercial or financial relationships that could be construed as a potential conflict of interest.

## References

[B1] Armitage P. Berry G. Matthews J. N. S. (2002). *Statistical Methods in Medical Research*, 4th Edn. Malden, MA: Blackwell Science 10.1002/9780470773666

[B2] Baddeley A. D. Hitch G. J. (1974). Working memory. *Psychol. Learn. Motiv.* 8 47–89. 10.1016/S0079-7421(08)60452-1

[B3] Baddeley A. D. Logie R. Nimmo-Smith I. Brereton N. (1985). Components of fluent reading. *J. Mem. Lang.* 24 119–131. 10.1016/0749-596X(85)90019-1

[B4] Bays P. M. Catalao R. F. G. Husain M. (2009). The precision of visual working memory is set by allocation of a shared resource. *J. Vis.* 9 1–11. 10.1167/9.10.7 19810788 PMC3118422

[B5] Bays P. M. Husain M. (2008). Dynamic shifts of limited working memory resources in human vision. *Science* 321 851–854. 10.1126/science.1158023 18687968 PMC2532743

[B6] Brainard D. H. (1997). The psychophysics toolbox. *Spat. Vis.* 10 433–436. 10.1163/156856897X00357 9176952

[B7] Burton A. M. White D. McNeill A. (2010). The Glasgow face matching test. *Behav. Res. Methods* 42 286–291. 10.3758/BRM.42.1.286 20160307

[B8] Cowan N. (2001). The magical number 4 in short-term memory: a reconsideration of mental storage capacity. *Behav. Brain Sci.* 24 87–185. 10.1017/S0140525X01003922 11515286

[B9] Grill-Spector K. Malach R. (2004). The human visual cortex. *Annu. Rev. Neurosci.* 27 649–677. 10.1146/annurev.neuro.27.070203.144220 15217346

[B10] Harrison S. A. Tong F. (2009). Decoding reveals the contents of visual working memory in early visual areas. *Nature* 458 632–635. 10.1038/nature07832 19225460 PMC2709809

[B11] Hayhoe M. M. Shrivastava A. Mruczek R. Pelz J. B. (2003). Visual memory and motor planning in a natural task. *J. Vis.* 3 49–63. 10.1167/3.1.6 12678625

[B12] Hitch G. J. Baddeley A. D. (1976). Verbal reasoning and working memory. *Q. J. Exp. Psychol.* 28 603–621. 10.1080/14640747608400587

[B13] Hollingworth A. Richard A. M. Luck S. J. (2008). Understanding the function of visual short-term memory: transsaccadic memory, object correspondence, and gaze correction. *J. Exp. Psychol. Gen.* 137 163–181. 10.1037/0096-3445.137.1.163 18248135 PMC2784885

[B14] Jiang Y. V. Shim W. M. Makovski T. (2008). Visual working memory for line orientations and face identities. *Percept. Psychophys.* 70 1581–1591. 10.3758/PP.70.8.1581 19064500 PMC2699186

[B15] Liang Y. Pertzov Y. Nicholas J. M. Henley S. M. D. Crutch S. Woodward F. (2016). Visual short-term memory binding deficit in familial Alzheimer’s disease. *Cortex* 78 150–164. 10.1016/j.cortex.2016.01.015 27085491 PMC4865502

[B16] Lorenc E. S. Pratte M. S. Angeloni C. F. Tong F. (2014). Expertise for upright faces improves the precision but not the capacity of visual working memory. *Atten. Percept. Psychophys.* 76 1975–1984. 10.3758/s13414-014-0653-z 24627213 PMC4163543

[B17] Love J. Selker R. Verhagen J. Marsman M. Gronau Q. F. Jamil T. (2015). *JASP (Version 0.6) [Computer software]*.

[B18] Luck S. J. Vogel E. K. (1997). The capacity of visual working memory for features and conjunctions. *Nature* 390 279–281. 10.1038/36846 9384378

[B19] Luck S. J. Vogel E. K. (2013). Visual working memory capacity: from psychophysics and neurobiology to individual differences. *Trends Cogn. Sci.* 17 391–400. 10.1016/j.tics.2013.06.006 23850263 PMC3729738

[B20] Ma W. J. (2018). Problematic usage of the Zhang and Luck mixture model. *Biorxiv* [Preprint]. 10.1101/268961

[B21] Ma W. J. Husain M. Bays P. M. (2014). Changing concepts of working memory. *Nat. Neurosci.* 17 347–356. 10.1038/nn.3655 24569831 PMC4159388

[B22] Magnussen S. Greenlee M. W. Thomas J. P. (1996). Parallel processing in visual short-term memory. *J. Exp. Psychol. Hum. Percept. Perform.* 22 202–212. 10.1037/0096-1523.22.1.202 8742262

[B23] Minear M. Park D. C. (2004). A lifespan database of adult facial stimuli. *Behav. Res. Methods Instrum. Comput.* 36 630–633. 10.3758/BF03206543 15641408

[B24] Morey R. D. Rouder J. N. (2015). *BayesFactor (Version 0.9.10-2)* [*Computer software*].

[B25] Nordstrøm M. M. Larsen M. Sierakowski J. Stegmann M. B. (2004). *The IMM Face Database - An Annotated Dataset of 240 Face Images.* Denmark: Technical University of Denmark.

[B26] Pelli D. G. (1997). The VideoToolbox software for visual psychophysics: transforming numbers into movies. *Spat. Vis.* 10 437–442. 10.1163/156856897X00366 9176953

[B27] Pertzov Y. Bays P. M. Joseph S. Husain M. (2013). Rapid forgetting prevented by retrospective attention cues. *J. Exp. Psychol. Hum. Percept. Perform.* 39 1224–1231. 10.1037/a0030947 23244045 PMC3793901

[B28] Pertzov Y. Dong M. Y. Peich M. C. Husain M. (2012). Forgetting what was where: the fragility of object-location binding. *PLoS One* 7:e48214. 10.1371/journal.pone.0048214 23118956 PMC3485137

[B29] Pertzov Y. Husain M. (2013). The privileged role of location in visual working memory. *Atten. Percept. Psychophys.* 76 1914–1924. 10.3758/s13414-013-0541-y 24027033 PMC4212176

[B30] Pertzov Y. Manohar S. Husain M. (2016). Rapid forgetting results from competition over time between items in visual working memory. *J. Exp. Psychol. Learn. Mem. Cogn.* 43 528–536. 10.1037/xlm0000328 27668485 PMC5377990

[B31] Pertzov Y. Manohar S. Husain M. (2017). Rapid forgetting results from competition over time between items in visual working memory. *J. Exp. Psychol. Learn. Mem. Cogn.* 43 528–536. 10.1037/xlm0000328 27668485 PMC5377990

[B32] Phillips W. A. (1974). On the distinction between sensory storage and short-term visual memory. *Percept. Psychophys.* 16 283–290. 10.3758/BF03203943

[B33] Prinzmetal W. Amiri H. Allen K. Edwards T. (1998). Phenomenology of attention: 1. *J. Exp. Psychol. Hum. Percept. Perform.* 24 261–282. 10.1037/0096-1523.24.1.261

[B34] Regan D. Beverley K. I. (1985). Postadaptation orientation discrimination. *J. Opt. Soc. Am. A* 2 147–155. 10.1364/JOSAA.2.000147 3973752

[B35] Richler J. J. Gauthier I. (2014). A meta-analysis and review of holistic face processing. *Psychol. Bull.* 140 1281–1302. 10.1037/a0037004 24956123 PMC4152424

[B36] Ricker T. J. Cowan N. (2010). Loss of visual working memory within seconds: the combined use of refreshable and non-refreshable features. *J. Exp. Psychol. Learn. Mem. Cogn.* 36 1355–1368. 10.1037/a0020356 20804281 PMC2970679

[B37] Ricker T. J. Cowan N. (2014). Differences between presentation methods in working memory procedures. *J. Exp. Psychol. Learn. Mem. Cogn.* 40 417–428. 10.1037/a0034301 24059859 PMC4056671

[B38] Rossion B. (2013). The composite face illusion: a whole window into our understanding of holistic face perception. *Vis. Cogn.* 21 139–253. 10.1080/13506285.2013.772929

[B39] Rossion B. Gauthier I. (2002). How does the brain process upright and inverted faces? *Behav. Cogn. Neurosci. Rev.* 1 62–74. 10.1177/1534582302001001004 17715586

[B40] Rouder J. N. Morey R. D. Speckman P. L. Province J. M. (2012). Default Bayes factors for ANOVA designs. *J. Math. Psychol.* 56 356–374. 10.1016/j.jmp.2012.08.001

[B41] Simons D. J. (1996). In sight, out of mind: when object representaions fail. *Psychol. Sci.* 7 301–305. 10.1111/j.1467-9280.1996.tb00378.x

[B42] Smith S. M. Vela E. (2001). Environmental context-dependent memory: a review and meta-analysis. *Psychon. Bull. Rev.* 8 203–220. 10.3758/BF03196157 11495110

[B43] Tong F. (2003). Primary visual cortex and visual awareness. *Nat. Rev. Neurosci.* 4 219–229. 10.1038/nrn1055 12612634

[B44] Wilken P. Ma W. J. (2004). A detection theory account of change detection. *J. Vis.* 4 1120–1135. 10.1167/4.12.11 15669916

[B45] Yin R. K. (1969). Looking at upside-down faces. *J. Exp. Psychol.* 81 141–145. 10.1037/h0027474

[B46] Zhang W. Luck S. J. (2008). Discrete fixed-resolution representations in visual working memory. *Nature* 453 233–236. 10.1038/nature06860 18385672 PMC2588137

[B47] Zhang W. Luck S. J. (2009). Sudden death and gradual decay in visual working memory. *Psychol. Sci.* 20 423–428. 10.1111/j.1467-9280.2009.02322.x 19320861 PMC2672970

[B48] Zhou X. Mondloch C. J. Emrich S. M. (2018). Encoding differences affect the number and precision of own- vs. other-race faces stored in visual working memory. *Atten. Percept. Psychophys.* 80 702–712. 10.1002/14356007.a13 29344908 PMC5838204

