# Supplementary material for: The Rapid Forgetting of Faces
Source: Front Psychol. 2018 Jul 27;9:1319. doi: 10.3389/fpsyg.2018.01319 (PMC6074000; doi:10.3389/fpsyg.2018.01319)
Supplement: Supplementary file 1 [file Data_Sheet_1.docx]

**SUPPLEMENTARY MATERIALS**

***Supplementary material* *– 1* – A JZS Bayes factor ANOVA for the three experiments.**

For each analysis the main text includes the two models with the highest posterior probability and the probability ratio between them. Here we provide the BF results for all models.

**Table 1.** Bayes factor (BF) of each tested model in Experiment 1 – for random and precision errors separately.

|  | **Random errors** | **Precision errors** |
| --- | --- | --- |
| **Models** | **BF _M_** | **BF _M_** |
| Null model (incl. subject) | 5.681e -4 | 4.117 |
| Delay duration | 7.461e -4 | 0.819 |
| Set size | 0.756 | 1.117 |
| Delay duration + Set size | 5.98 | 0.336 |
| Delay duration + Set size + Delay duration ✻ Set size | 1.274 | 0.111 |

**Table 2**. Bayes factor (BF) of each tested model in Experiment 2 – for random and precision errors separately. For greater readability, the effects of experiment type are not fully reported in the table (but this factor was included in the Bayes factor ANOVA).

|  | **Random errors** | **Precision errors** |
| --- | --- | --- |
| **Models** | **BF _M_** | **BF _M_** |
| Null model (incl. subject) | 8.415e -9 | 0.005 |
| Delay duration | 6.783e -9 | 0.001 |
| Face orientation | 3.197 | 17.421 |
| Delay duration + Face orientation | 5.855 | 1.93 |
| Delay duration + Face orientation + Delay duration ✻ Face orientation | 3.199 | 0.914 |
| Different models that include Experiment type | ≤ 1.378 | ≤ 3.441 |

**Table 3**. Bayes factor (BF) of each tested model in Experiment 3 – for random and precision errors separately. In addition, for precision errors we provide the BF without the simultaneous perception condition (only 1 and 6 second delays included in the delay duration factor).

|  | **Random errors** | **Precision errors** | **Precision errors (without the perception condition)** |
| --- | --- | --- | --- |
| **Models** | **BF _M_** | **BF _M_** | **BF _M_** |
| Null model (incl. subject) | 7.980e -9 | 1.263e -15 | 9.948e -6 |
| Delay duration | 0.149 | 3.158e  -8 | 2.640e -6 |
| Face orientation | 3.590e -8 | 5.162e -11 | 9.693 |
| Delay duration + Face orientation | 20.471 | 14.614 | 1.014 |
| Delay duration + Face orientation + Delay duration ✻ Face orientation | 0.584 | 1.095 | 0.395 |

***Supplementary material* *– 2 –* Data analysis for Experiment 1 with the previously excluded face condition (with the 3 displayed faces in the report cycle).**

In Experiment 1 there were two types of trials with three faces in the memory array. The main text only discusses the type in which the 18 face report circle was composed of the target face and 2 novel faces. The second type, in which the three faces from the memory array composed the report circle, were excluded from the analysis in the main text, because the distribution of random errors was not uniform (unlike in the other conditions). Here we report the analysis that also includes the second type. The analysis techniques are the same as in the main text, but this time the '3 face' condition include the two types.

For the statistical analysis, we applied a repeated measures ANOVA with number of faces (1 or 3) and delay duration (1 or 6 seconds) as factors. In the current analysis, the 3 face condition included the two types of trials with 3 faces in the memory array. The proportion of random errors and the averaged precision errors were the dependent variables (two different ANOVAs).

**Random errors –** A repeated measures ANOVA showed that the proportion of random errors descriptive increased with delay duration [Delay main effect: F(1, 11) = 4.628, p = 0.055, ɳ^2^ = 0.296], and increased with the increase in set size [Set size main effect: F(1,11) = 17.079, p = 0.002, ɳ^2^ = 0.608]; but with no significant interaction [interaction: F(1,11) = 0.184, p = 0.676, ɳ^2^ = 0.016].

**Precision errors** – A repeated measures ANOVA showed no effect of delay duration or set size, or interaction [Main effect set size: F(1,11) = 0.686, p = 0.425, ɳ^2^ = 0.059. Delay duration effect F(1,11) = 0.765, p = 0.400, ɳ^2^ = 0.065. Interaction: F(1, 11) = 0.170, p = 0.688, ɳ^2^ = 0.015].
